# Vapour-phase effects of thyme and eucalyptus oils, alone and combined, against toxin-producing fungi from stored durum wheat

**DOI:** 10.1038/s41598-025-21379-7

**Published:** 2025-12-27

**Authors:** Amine Bousta, Salma Astati, Imane Wahby, Abdelwahed Fidah, Tarik Janah, Mohamed Rahouti, Laila Rhazi

**Affiliations:** 1 Research Center of Plant and Microbial Biotechnologies, Biodiversity and Environment, Laboratory of Botany and Valorisation of Plant and Fungal Resources, Department of Biology, Faculty of Sciences, Mohammed V University , Rabat, Morocco B.P. 1014 RP, 4 Avenue Ibn Battouta, 10000; 2 Laboratory of Photochemistry, Office of Wood Technology and Forest Products Valorization, Forest Research Center (CRF), Rabat, Morocco Agdal, B.P. 763, Av. Omar Ibn Al Khattab, 10050; 3 Research and Training Innovation Center (CIRF), National Agency for Water and Forests (ANEF), 10000, Av. Mohammed VI, Rabat, Morocco

**Keywords:** Biological techniques, Biotechnology

## Abstract

The food system is dependent on durum wheat (*Triticum durum*), which is considered one of the most important agricultural elements. Food security is ensured by a sustainable protection strategy against harmful microorganisms, such as fungi. The broad-spectrum antifungal activity of EOs against most problematic storage fungi has been demonstrated. The antifungal properties of EOs derived from *Thymus satureioides* and *Eucalyptus camaldulensis* were evaluated in vitro. The diverse biological functions of these plants are world-renowned. The microatmosphere technique was used to test antifungal activity against four postharvest pathogens associated with durum wheat in storage: *Aspergillus aflatoxiformans*,* Aspergillus flavus*,* Aspergillus nidulans*,* Aspergillus versicolor*. The potential synergy of combining *T. satureioides* and *E. camaldulensis* was explored in this study. The results showed that *T. satureioides* leaf EO was the most effective, with MIC values ranging from 0.025 to 0.1 µL/ml. The high content of isoborneol (17.87%) in this oil was identified as a significant contributor to its potent antifungal properties. The least effective vapor against all tested pathogens was produced by the individual application of *Eucalyptus* oil (MIC 0.05–0.31 µL/ml). The combination of *Thyme* and *Eucalyptus* leaf EOs showed partial synergy against all tested pathogens (FICI values between 0.50 and 0.75) and fully synergistic against *A. nidulans*. The production of aflatoxin B1 (AFB1) and ochratoxine A (OTA) was inhibited after treatment with MICs and 2xMICs of oils alone, a concentration below the MIC, indicating weak anti-ochra/aflatoxigenic effect. The potential for the development of specific formulations aimed at enhancing grain protection during storage is evident in this synergistic combination.

## Introduction

Durum wheat and common wheat are the most commonly used and consumed crops in the cultivation of cereals worldwide. Based on the literature data, the global mycotoxin prevalence in food crops varies largely depending on many factors, such as the mycotoxin of concern, used analytical methods and reporting of the results, but it appears that the prevalence for the detected mycotoxins is up to 60–80%^1^. Mycotoxins are toxic chemical products formed as secondary metabolites by a few fungal species that colonize crops and contaminate them with toxins in the field or after harvest. They are produced when micro-ecological conditions are favourable for the growth and multiplication of fungi. The main toxic compounds associated with the contamination include deoxynivalenol (or nivalenol in some areas), fumonisins, zearalenone, aflatoxins, and ochratoxin A. These are the most important mycotoxins produced by species of *Aspergillus*, *Fusarium*, and *Penicillium *^2^. Furthermore, most of the mycotoxins are known to be chemically stable, with pre- and post-harvest mitigation strategies, such as with food and feed processing, only eliminating them to a certain extent^1^. The search for biological antifungal agents to replace synthetic pesticides has grown in recent years. Among natural antimicrobial products, especially interesting are plant products such as Essential Oils (EOs)^3^. An EO’s strong fungicidal effects^4^ make them an important seed-decontamination alternative to synthetic seed preservers. For active packaging and fumigation of storage crops, this would be a volatile screening method. Alternatively, there are a limited number of research studies reported in the current literature on the application of EOs based microemulsion for antimicrobial activity^5^. Therefore, the study will contribute to developing safer natural products and an effective strategy to control the growth of ochratoxigenic moulds and ochratoxin contamination^6^. As a result, the objective of this study was to evaluate the in vitro effectiveness of *Thymus satureoides* and *Eucalyptus camaldulensis* EO vapours applied both individually and in combination to inhibit the mycelial growth of *A. flavus*, *A. aflatoxiformans*, *A. nidulans*, *A. versicolor* and their secretion of aflatoxine B1 (AFB1) and ochratoxine A (OTA).

## Materials and methods

### Identification of fungal cultures isolated from durum wheat

The fungal strains used in present study were isolated in our laboratory from grain samples of wheat (*Triticum durum*). Identification of the four chosen isolates was based on the 5.8 S-Internal Transcribed Spacer (ITS) region. Thus, a set of primers (ITS1 and ITS4) was used to amplify the recombinant deoxyribonucleic acid (rDNA) region, which includes the two-non-coding ITS1 and ITS2 segments and the 5.8 Ribosomal ribonucleic acid (rRNA) gene. Our BLAST data showed that the isolates were related to *A. aflatoxiformans* (OR651258), *A. flavus* (OR651255), *A. nidulans* (OR651269), *A. versicolor* (OR651271)^7^.

The macroscopic appearance of *A. flavus* colonies is typically flat, granular, and velvety to powdery in texture, with yellow-green to olive-green coloration and a yellowish to light brown reverse; it grows rapidly on common media. *A. aflatoxiformans* closely resemble *A. flavus* but tends to have slightly darker green colonies and a cream to pale brown reverse, also exhibiting moderately rapid growth. *A. nidulans* forms dense, fuzzy colonies with dark green to olive coloration, often displaying red or violet pigmentation on the reverse due to secondary metabolites; its growth is rapid. *A. versicolor* displays flat, often wrinkled or zoned colonies with a wide color range from pale green to blue-green, frequently with cream, yellow, or orange hues; the reverse is usually colorless to light brown, and the colony grows slowly to moderately.

### Plant materials

In Morocco, two plants belonging to the *Lamiaceae* and *Myrtaceae* plant families were harvested in their natural habitats during the flowering season (Table 1). Plant materials used in this study, including *Thymus satureioides* and *Eucalyptus camaldulensis*, were collected in accordance with relevant regulations. Necessary permissions and/or collection licenses were obtained from the appropriate authorities prior to sampling. The formal identification of the plant species was carried out by a qualified taxonomist, Professor R. Laila, in the national herbarium in the faculty of sciences, university Mohamed V in Rabat—Morocco, where voucher specimens were kept. (Voucher numbers: *E. camaldulensis*: 264 and *T. satureioides*: 265).

**Table 1:** Aromatic and medicinal plant species investigated in this study.

Species name	Family	Parts used	Harvesting location	Altitude (M)	Harvesting season
*Thymus satureioides*	*Lamiaceae*	Leaves	Souss-Massa Region	1020	May–June/2021
*Eucalyptus camaldulensis*	*Myrtaceae*		Rabat-Salé-Kenitra Region	< 300 M	Novembre– Décembre/2021

### Extraction and analysis of EOs by GC/MS

All leaf biomass extract samples were subjected to hydrodistillation by a Clevenger type apparatus at the Laboratory of phytochemistry, Department of Wood Technology and Forest Products Valorization, National Agency for Water and Forests (ANEF), Rabat, Morocco according to the method of^8^.The EOs were analyzed using a Hewlett Packard 6890 GC equipped with a flame ionization detector (FID) and a DB-5 (30 m × 0.25 mm i.d., film thickness 0.25 μm) capillary column was used. The column and analysis conditions were the same as in GC-MS, as expressed below. The percentage composition of the EOs was computed from GC-FID peak areas without correction factors^9^.

### Fungal growth inhibition assay using single EOs

The assay was carried out in a Petrie dish containing CYA medium. The fungal spore inoculums (concentration 10^6^ spores per ml) of chosen mycotoxigenic fungi were applied to the CYA surface after being prepared. The lid of each Petrie dish was covered in a sterile filter paper disc with different EO volume with water added to for the nil control treatment. The Petrie dishes were sealed and incubated upside down for 7 days at 32 °C. The study was conducted with three replications for each treatment. After 7 days of incubation, the antifungal activity of each EO was evaluated by measuring the colony diameter twice diagonally. The percentage inhibition of mycelial growth was determined using the proposed formula by^10^:

$$\:Percent\:Inhibition=\frac{{M}_{c}-Mt}{Mc}\times\:100$$

where Mc is the averaged colony diameter (mm) for control. Mt is the averaged colony diameter (mm) for treatment.

### Minimum inhibitory concentration (MIC)

The antifungal activity of the EO vapor doses in the screening test was demonstrated by serial dilution of them at 75%, 50%, and 25% corresponding to proportional volumes of EO applied on the filter paper. For example, to obtain 50% of the maximum concentration, half the volume of EO used for the 100% concentration was applied. Thus, the dilution was performed by directly adjusting the volume of EO deposited, without using any solvent. The MIC was considered to be the lowest concentration of EO vapours preventing colony formation^11^.

MIC = minimum inhibitory dose (µL)/ olume of air above the oil in Petrie dish (ml).

The tested concentrations (75%, 50%, and 25%).

### Antifungal assay with combination of EOs

A checkerboard assay was used to evaluate combinations of EOs for their synergistic inhibitory activity against fungus. Concentrations of 1 MIC to 1/4 MIC of each EOs were added to sterile filter discs, placed in the center of the lid of Petri dishes to share the same atmosphere. The plates were incubated at 30 °C for seven days. Confirming and verifying the occurrence of synergistic antifungal activity was accomplished by calculating the fractional inhibitory concentration (FIC) and fractional inhibitory concentration index (FICI). A combination with a FICI value ≤ 0.5 was considered to have a synergistic effect, an additive effect if 0.5 < FICI ≤ 1, an indifferent effect when 1 < FICI < 2 and an antagonistic effect when FICI ≥ 2.

For each replicate, FICI values were calculated using the following formula:

$$\:\mathrm{FICI}={\mathrm{FIC}}_{\mathrm{Thyme}\mathrm{\:EO}}+{\mathrm{FIC}}_{\mathrm{Eucalyptus}\mathrm{\:EO}}$$

The FIC is the MIC of EO in combination/MIC for EO alone^12^.

### Evaluation of the fungistatic/fungicidal effect of the EOs

The antimicrobial atmosphere created when the filter disk and cover were replaced with another sterile cover resulted in static or cidal effects. If microorganisms start growing after removal, there is a static effect, while there is a cidal effect if no growth occurs^13^.

### The efficacy of plant EOs against fungal production of AFB1 and OTA

The MICs obtained in the antifungal assay were used in both cases of individual, making the MIC diluted and increased once to get three treatments for each EO, such as 1/2MIC, MIC, and 2MIC. The inhibition of mycelial growth by EOs does not necessarily correspond to mycotoxines inhibition^11^. A previously described HPLC extraction method was used to evaluate isolates belonging to fungi and purified according to the methods of^14, 15, 16^. µg per g of CYA media was used to express the mycotoxin producing capacity. The percentage of inhibition of mycotoxin production was calculated for every treatment in relation to the control set^17^. After extraction, AFs measurements were performed by HPLC-FD.

### Statistical analysis of data

Data were expressed as means ± standard deviation (*n* = 3). A one-way analysis of variance (ANOVA) was performed to compare the minimum inhibitory concentrations (MICs) of the essential oils used individually across the different fungal strains. When the ANOVA indicated significant differences (*p* < 0.05), Tukey’s post-hoc test was applied to identify statistically distinct groups. To evaluate the effects of essential oil combinations, a separate one-way ANOVA was conducted for each fungal strain, comparing the MICs of individual oils versus those in combination. Differences were considered statistically significant at *p* < 0.05. All statistical analyses were performed using SPSS software version 31^18^. All experiments were performed in triplicate, and both positive and negative controls were included for comparative purposes.

## Results and discussion

### EOs yield and composition discussion

The principal component of *Eucalyptus* essential oil is 1,8-cineole, a terpene oxide^19^. The *Eucalyptus* genus, native to Australia and Tasmania, comprises more than 800 species and has successfully spread worldwide due to its high adaptability and rapid growth^20^. Consequently, *Eucalyptus* has become one of the most widely planted genera globally^21^.

In our study, 36 compounds were identified in the leaf essential oil (EO) of *E. camaldulensis* (Table 2). The predominant constituents were 1,8-cineole (55.13%), 1,3,8-p-menthatriene (9.13%), and α-pinene (4.93%). At the national level, these findings are consistent with those reported for *E. camaldulensis* from the Sidi Amira region in Morocco, where 1,8-cineole was also identified as the major compound^22^.

However, lower percentages of 1,8-cineole were recorded in other Moroccan regions. In the Khouribga region, the EO of *E. camaldulensis* contained 34.22% 1,8-cineole^23^, while in the Taounate region, only 19.05% was observed^24^. Additionally, a sample collected from the same location (unspecified) exhibited a markedly different chemical profile, with a yield of 0.75%. In this case, the dominant compounds were p-cymene (35.11%), γ-eudesmol (11.90%), L-linalool (11.51%), and piperitone (10.28%)^25^.

About 45 constituents were identified in EO of *T. satureioides* in this study. major components are isoborneol 17.87%, thymol 17.78% and camphene 8.33%. Other compounds having contents of less than 8%, were also found in these EO, such as Thymol methyl ether (7.90%), α-Pinene (5.76%) and p-Cymene 5.58%. Our results are approximatively in accordance with those found by other authors from Morocco. Leaves of *T. satureioides* originated from southwest Morocco gave yields of EO varying between 1.35 and 2.32% depending on the *thyme* population. Oulad Berhil population gave the best yield while that of Timoulay Aksri yielded the lowest amount of EOs, borneol as major compound 33.03% (aoulouz) and 40.11% (ouled berhil)^19^. Thirty-two samples wild *T. satureioides* were collected from High Atlas Mountains (Valley of Agoundis). The yield of EOs ranged from 0.2 to 2.3%. The predominant compound was borneol, (22.67–37.47%)^20^, . The yield increases with phenological stages to reach the maximum value at the flowering and fruiting periods according to harvest sites^21^. From northern Morocco, major ones are endo-borneol (22.73%), carvacrol (16.96%), and α-terpineol (12.20%)^22^. Contrary, and from the same location GC/MS of *T. satureioides* EO collected from Al Haouz province, lead to the identification of thymol (28.66%) followed by borneol (21.16%)^23^.

**Table 2:** :Components identified in the EOs.

Component name	KI	Percentage of identified compound in isolated EO	
		Thymus satureioides	Eucalyptus camaldulensis
α-thujene	0931	**1.488**	0.384
α-pinene	0939	**5.763**	**4.939**
Camphene	0953	**8.336**	0.113
β-pinene	0980	1.597	0.176
Myrcene	0991	**1.409**	0.502
P-cymene	1026	**5.584**	**2.518**
1,3,8-p-menthatriène	1111	–	**9.134**
-D-limonene	1031	**1.375**	–
Eucalyptol	1033	0.073	**55.138**
(Z)-β-Ocimene	1040	0.038	**3.890**
γ- terpinene	1062	**3.993**	**3.050**
Linalool	1098	**4.395**	0.601
Isoborneol	1156	**17.878**	–
Terpinene-4-ol	1177	**2.381**	**2.159**
γ-terpineol	1189	**4.213**	**3.192**
Thymol methyl ether	1235	**7.902**	–
Thymol	**1290**	**17.78**	0.149
E-caryophyllene	1418	**4.052**	–
Spathulenol	1576	–	**3.739**
Globulol	1583	–	**1.537**
Yield (%)		2.39	0.94
Chemotype		**Iso bornéol /** **Thymol/ Camphene**	**Eucalyptol**

### Effects of EOs volatiles on fungal growth

#### Antifungal activity with single EOs

Tukey’s post-hoc test confirmed that these differences were statistically significant for most of the combinations compared. Significant differences in terms of efficacy were seen among individual EOs. Results showed that all EOs had an inhibitory activity against fungal mycelial growth (Table 3). *T. satureoides* EO was the most effective against all pathogens at all levels of dosage. At 5 µL it completely inhibited *A. flavus*,* A. nidulans*, and *A. versicolor* instead it wasn’t effective against, *A. aflatoxiformans*, 10 µL was the dose required for the inhibition of that fungus. While *E. camaldulensis* EO vapour totally inhibited all pathogens at 50 µl except for *A. aflatoxiformans* (96.26%). With the above results, MIC (vol/vol of air) of each EO were determined and presented in Table 3. *T. satureoides* oil showed the lowest MICs values (0.025–0.10 µL/ml), while the MIC of *E. camaldulensis* oil ranged between (0.05–0.31 µL/ml) for all pathogens. The difference in the MICs could be attributed to the difference in fungal strains used in this work, which exhibited more resistance, as well as the chemical constituents of the EO.

The antifungal activity of *E. camaldulensis* EO is primarily attributed to its high 1,8-cineole content, although this compound alone has only limited antifungal effects. The overall antimicrobial efficacy of EOs is determined not only by their major constituents but also by synergistic interactions between their various components^24^. The antifungal mechanism of *E. camaldulensis* remains partially understood and warrants further investigation^25^.

In vapor-phase antifungal assays, textile samples treated with *Eucalyptus camaldulensis* essential oil (EO) showed strong activity. *Aspergillus flavus* and *A. niger* exhibited no visible growth after 7 and 14 days of incubation at 25 ± 1 °C on PDA medium containing 0.5 µL/mL of EO^26^. In contrast, higher MIC values were reported in a previous study^11^ higher MIC value—above 0.74, 0.56, and 0.56 µL/mL of air—for *A. niger*, *A. flavus*, and *Fusarium proliferatum*, respectively. These values are approximately twice as high as those found in our experiments, suggesting improved efficacy under the current conditions.

Other works have shown a wide range of MICs depending on the methodology used. For instance, in a study employing a modified macro-broth dilution method^27^, a concentration of 125 µL/mL completely inhibited *A. flavus* and *A. niger* at 25 °C over 7 days. Similarly, MICs of 12 µL/mL and 7 µL/mL for *A. flavus* and *A. niger*, respectively, were reported on PDA medium incubated at 26 ± 1 °C for 14 days^28^. In another study, antifungal effects of *E. camaldulensis* EO were observed with MICs of 0.47 mg/mL for *A. niger* and 0.43 mg/mL for *A. flavus*^29^. These differences in MIC values may reflect variations in EO composition due to geographic origin, fungal strain susceptibility, and experimental design. Mechanistically, several studies suggest that the antifungal action of EOs involves disruption of the fungal plasma membrane and induction of mitochondrial dysfunction, leading to the accumulation of reactive oxygen species (ROS) and ultimately cell death^26, 27^.

Comparatively, *Thymus* and *Origanum* essential oils (EOs) have shown strong antifungal activity via vapor contact assays. In one study^28^, thyme and oregano EOs completely inhibited fungal growth (100%) on CYA medium at 0.5 µL/mL of air after 14 days of incubation with *A. flavus*, *A. parasiticus*, and *A. westerdijkiae*. In another investigation^29^, linen samples infected with *A. flavus* (10 µL/mL) fully recovered after 8 weeks of exposure to thyme EO, while complete eradication on papyrus samples occurred within just one week. These higher MIC values, compared to our results, emphasize the strong potential of *E. camaldulensis* EO. Limited inhibition on PDA medium after 7 days was observed in a separate study^12^, with complete inhibition of *A. flavus* only at 25 µL per disc. In a related experiment, Guynot et al. reported total inhibition using 50 µL per filter paper in Petri dishes after 42 days of incubation on wheat flour agar, regardless of water activity (aw). Beyond the *Thymus* genus, *Monarda didyma* L., a Lamiaceae species rich in thymoquinone (36.02%), also exhibited antifungal activity, with MICs of 2% v/v (vapor phase) and 6% v/v (broth dilution) against *A. flavus*^30^.

Overall, these findings highlight the variability of EO antifungal activity depending on the plant species, concentration, fungal strain, and application method. Notably, our results demonstrate that *E. camaldulensis* EO exhibits a comparatively strong antifungal effect at relatively low concentrations, particularly in vapor form. These observations reinforce their potential as a natural antifungal agent, though further studies are needed to fully elucidate its mechanism of action and evaluate its practical applications in food preservation and textile protection.

**Table 3:** Evaluation of MIC and MFC of EOs against aflatoxigenic fungi species.

EOs	MIC (µL/ml of air) : Means ± Standard deviation(*n* = 3)			
	*A. aflatoxiformans*	*A. flavus*	*A. nidulans*	*A. versicolor*
*E. camaludelensis*	0.31 ± 0.01	0.25 ± 0.01	0.15 ± 0.01	0.05 ± 0.01
State	Fungistatic	Fungistatic	Fungistatic	Fungistatic
*T. satureioides*	0.10 ± 0.01	0.025 ± 0.005	0.075 ± 0.005	0.075 ± 0.005
State	Fungicide	Fungicide	Fungicide	Fungicide

#### EOs in combination

The combination of *Eucalyptus* and *Thymus* EOs exhibited a synergistic or partially synergistic antifungal effect against *Aspergillus* species. Specifically, the growth of *A. aflatoxiformans*,* A. flavus*, and *A. versicolor* was partially suppressed, while the effect against *A. nidulans* was fully synergistic. These combinations enabled a significant reduction in the required concentrations of individual EOs. Notably, the combination allowed a reduction of up to ¾ and ½ of the MICs of thyme and eucalyptus oils, respectively, for *A. aflatoxiformans* and *A. versicolor*. Similarly, for *A. nidulans*, the combination was effective at only ¾ MIC of each EO, and for A. flavus, at ½ MIC of thyme and ¾ MIC of eucalyptus oil (Table 4).

MICs of individual and combined EOs were statistically compared using one-way ANOVA (*n* = 3), revealing highly significant differences (*p* < 0.000001) for all tested fungal species. These findings confirm the synergistic or partially synergistic interactions between the two EOs.

In support of these results, strong antifungal activity of thymol and eucalyptol—major constituents of thyme and eucalyptus oils—against mycotoxigenic fungi such as *Fusarium* and *Aspergillus spp*. has been reported^3^. The synergistic mechanism is believed to involve increased membrane permeability and disruption of ergosterol synthesis in fungal cells, thereby enhancing their susceptibility to antifungal agents^28^.

**Table 4:** Fractional inhibitory concentrations for individual EO vapours and the fractional inhibitory concentration indices for combinations of EO vapours against the study fungi calculated from the MICs.

Fungus tested	*T. satureioides* (µL/mL)	*E. camaldulensis* (µL/mL)	Total (µL/mL)	FICI	Observed effect	*p*-value (ANOVA)
*A. aflatoxiformans*	0.025	0.155	0.180	0.75	Partially synergistic	3.45 × 10^−9^
*A. flavus*	0.0125	0.0625	0.075	0.75	Partially synergistic	3.67 × 10^−10^
*A. nidulans*	0.01875	0.0375	0.05625	0.50	Synergistic	3.12 × 10^−7^
*A. versicolor*	0.01875	0.025	0.04375	0.75	Partially synergistic	2.49 × 10^−7^

#### Anti-aflatoxin efficacy of EO vapours

**Fig. 1:**
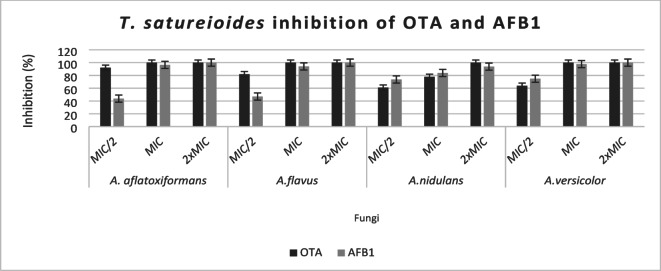
Effect of *T. satureioides* EO volatile on AFB1 and OTA accumulation by tested strains.

**Fig. 2:**
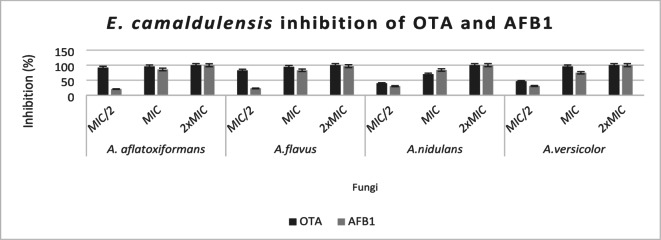
Effect of *E. camaldulensis* EO volatile on AFB1 and OTA accumulation by tested strains.

*T. satureioides* EO exhibited slightly higher antifungal and antimycotoxigenic efficacy compared to *E. camaldulensis* oil (Figs. 1 and 2). The inhibition of aflatoxin production was dose-dependent, with lower EO concentrations producing less pronounced effects. The production of AFB1 and OTA by fungi, which varies with both EO concentration and fungal strain, was completely inhibited at 2× MIC.

Aflatoxin B1 (AFB1) production by *A. flavus* grown on CYA medium was significantly reduced by *T. vulgaris* EO at 545.26 µg/mL, while aflatoxin G2 became undetectable at only 10.91 µg/mL, as shown in previous studies^15,30]^. The suppression of ochratoxin A (OTA) production has been genetically linked to the downregulation of key biosynthetic genes such as *acOTApks*, *acOTAnrps*, and *acpks*, as well as regulatory genes *veA* and *laeA*^29^.

The anti-aflatoxigenic activity of essential oils has also been associated with the inhibition of early steps in aflatoxin biosynthesis, particularly through interference with lipid peroxidation processes^31^. Furthermore, key enzymes involved in lipid and carbohydrate catabolism in mycotoxigenic fungi have been identified as potential targets of the antimycotoxigenic action of EOs^32^.

Our investigation has demonstrated that essential oils (EOs) derived from common aromatic plants possess significant potential as natural fumigants. This study confirmed that the antifungal activity of thyme and eucalyptus EOs is highly effective against toxigenic *Aspergillus* species in the vapor phase under in vitro conditions. These EOs exhibited strong inhibitory effects on fungal growth and mycotoxin production, with thyme and eucalyptus oils showing maximum efficacy at concentrations of 100 µL/L and 640 µL/L of air, respectively.

Given their pronounced antimycotoxigenic activity, these EOs could be considered promising candidates for controlling fungal contamination and mycotoxin accumulation, particularly aflatoxins and ochratoxins, in stored food and agricultural products. Furthermore, the use of EO combinations may enhance efficacy while reducing the required dosages, as demonstrated in our synergy assays. This strategy could offer a sustainable, residue-free alternative to synthetic fungicides.

Nonetheless, the successful application of EOs in real food systems depends on several factors, including the food matrix, storage conditions, EO volatility, and sensory compatibility. As highlighted optimizing the combination between specific EOs and targeted food products is crucial for practical implementation^30^.

## Data Availability

The datasets generated and/or analysed during the current study are not publicly available due to privacy, but are available from the corresponding author on reasonable request.
